# Remodeling in the AV block dog is essential for tolerating moderate treadmill activity^☆^

**DOI:** 10.1016/j.ijcha.2022.101169

**Published:** 2023-01-03

**Authors:** Joanne J.A. van Bavel, Henriëtte D.M. Beekman, Arend Schot, Philippe C. Wouters, Maarten G. van Emst, Tim Takken, Marcel A.G. van der Heyden, Marc A. Vos

**Affiliations:** a Department of Medical Physiology, University Medical Center Utrecht, Utrecht, the Netherlands; b Department of Clinical Sciences, Division of Anatomy and Physiology, Faculty of Veterinary Medicine, Utrecht University, the Netherlands; c Department of Cardiology, Division of Heart & Lungs, University Medical Center Utrecht, Utrecht, the Netherlands; d Child Development and Exercise Center, Wilhelmina Children’s Hospital, University Medical Center Utrecht, Utrecht, the Netherlands

**Keywords:** Exercise, AV block dog model, Remodeling, Cardiorespiratory changes, Treadmill

## Abstract

**Background:**

A preclinical model standardized at different remodeling stages after AV block induction in awake state is suitable for the evaluation of improved cardiac devices. We studied exercise-induced cardiorespiratory parameters at three different timepoints after inducing AV block in dogs.

**Methods:**

Mongrel dogs (n = 12) were placed on a treadmill with a 10% incline and performed a moderate exercise protocol (10-minute run at 6 km/h). Dogs ran at sinus rhythm (SR), at two days (AVB2d, initiation of remodeling), three weeks (CAVB3) and six weeks (CAVB6, completed remodeling) after AV block.

**Results:**

All dogs completed the exercise protocol at SR, CAVB3 and CAVB6, while 6/12 dogs at AVB2d failed to complete the exercise protocol. The atrial rate was higher at all AV block timepoints (126 ± 20 to 141 ± 19 bpm at rest and 221 ± 10 to 231 ± 13 bpm during exercise) compared to SR (100 ± 29 bpm at rest and 162 ± 28 bpm during exercise, p < 0.05). Upon exercise, stroke volume increased from 66 ± 15 ml at SR, to 96 ± 21 ml at AVB2d (p < 0.05), 91 ± 13 ml at CAVB3 (p < 0.05) and 85 ± 24 ml at CAVB6 but failed to compensate for the AV block-induced bradycardia. Therefore, cardiac output was lower after AV block compared to SR. Exercising dogs at AVB2d showed most arrhythmic events, lowest VO_2_, and signs of desaturation and acidification in venous blood.

**Conclusion:**

Dogs with limited remodeling after AV block have a reduced exercise tolerance, which is reflected in changes in cardiorespiratory parameters.

## 1 Introduction

Management of patients with bradycardia due to atrioventricular block (AV block) includes temporary pacing in the case of reversible causes (e.g., Lyme disease), medical therapy, and chronic therapy in the form of permanent pacing [1]. For young patients with congenital complete AV block (CCAVB), permanent pacing comes with therapeutic dilemmas regarding the decision at what age to implant the pacemaker, what type of pacing (location of pacing leads and mode of pacemaker) gains maximal benefits, and implantation of a device in patients that are asymptomatic [2]. Moreover, chronic pacing will invariably require the replacement of the leads and battery after a certain time period, and long-term side effects such as pacing-induced dyssynchrony resulting in adverse left ventricular remodeling [2]. In the current era of complex digital opportunities, including machine learning, there is high interest in improving cardiovascular device technologies based on physiological input.

Complex device technologies and algorithms developed over the last four decades focus on rate-adaptive pacing based on physiological input, including physical activity [3], transthoracic impedance [4], respiration [5], [6], and even a combination of various physiological parameters [7]. However, optimization of sensor sensitivity is challenging, and studies lack significant improvement outcome of the sensors on top of pacing at a fixed rate to treat bradycardia [8], [9]. Testing of new developed complex devices and algorithms for treatment of cardiorespiratory diseases by rate-adaptive pacing will benefit from a standardized preclinical model in which evaluation of diverse physiological parameters in conditions of rest and activity are incorporated.

The dog is described as the most predictive species in the cardiac electrophysiological research field [10]. The AV block dog model [11] was standardized over the last four decades as experimental model in pro- and antiarrhythmic drug screening [10] and testing of new device properties [12]. After induction of AV block, the acute bradycardia is accompanied with a decrease in cardiac output and therefore different adaptations are initiated in the subsequent weeks. These are 1) contractile remodeling which includes the enhancement of neurohormonal parameters to increase inotropy upon each cardiac contraction – initiated immediately and maximal after two weeks [13], 2) electrical remodeling in the form of diminished repolarizing ion currents I_Kr_ and I_Ks_ resulting in prolongation of the action potential reflected by QT prolongation – completed after two weeks [14], and 3) structural remodeling referring to the slower process of biventricular hypertrophy which takes over the increase in contractile force – stabilized after four to six weeks [15]. Despite the animal’s well-being in daily existence as model of compensated hypertrophy, dogs under anesthesia are sensitive to Torsade de Pointes arrhythmias after a dofetilide-trigger at > 2 weeks after AV block [16]. This model allows controlled evaluation of drug safety and device properties in anesthetized and awake conditions at different stages of remodeling.

In view of the necessity of a well characterized preclinical model for evaluating newly developed pacing technology, the aim of this study was to determine exercise tolerance and exercise-induced cardiorespiratory alterations in the moderately exercising AV block dog model without a pacing device at different remodeling stages after induction of AV block using minimally invasive techniques. It was hypothesized that dogs with remodeled hearts as induced by AV block have a better exercise performance than dogs in the first stage(s) of remodeling - with initiated but still limited adaptations – which would provide an optimal time window for future device testing.

## 2 Methods

### 2.1 Animals

Animal care and handling were in accordance with the ‘’Directive 2010/63/EU of the European Parliament and the Council of 22 September 2010 on the protection of animals used for scientific purposes’’ and the Dutch law as laid down in the Experiments on Animals Act. Experimental procedures were approved by the Central Authority for Scientific Procedures on Animals. Application approval numbers: AVD115002016531 and AVD11500202114910. Animal studies are reported in compliance with the ARRIVE guidelines [17]. The current study has no implications for replacement, refinement, or reduction.

Twelve purpose-bred mongrel dogs (Marshall, New York, USA) consisting of three females and nine males were included for serial experiments. They had a body weight of 26 ± 2 kg and were 13 ± 1 months old at the time of the first experiment. The animals were housed in pairs in kennels with wooden bedding material, had ad libitum access to water and received food pellets twice a day. They played outside once a day with access to playing toys, and their welfare was checked daily.

### 2.2 Surgical procedure

Animals were fasted during the night prior to the surgical procedure. Half an hour before the procedure, they received premedication (0.02 mg/kg i.m. atropine, 0.5 mg/kg i.m. methadone, 0.5 mg/kg i.m. acepromazine and 0.1 mg/kg s.c. meloxicam). General anesthesia was induced by sodium pentobarbital (pentobarbital, 25 mg/kg i.v.) and maintained by 1.5% isoflurane in O_2_ and N_2_O (1:2 ratio) via mechanical ventilation at 12 breaths/minute. Ampicillin (1000 mg) was administered before (i.v.) and after (i.m.) surgery, and buprenorphine (0.3 mg, i.m.) was provided after surgery. During the procedure, AV block was induced by radiofrequency ablation of the bundle of His [18]. A body temperature sensor (IPTT-300, Plexx BV, Elst, The Netherlands) was inserted subcutaneously in the neck of the dog under anesthesia.

### 2.3 Measurements

**Fig. 1:**
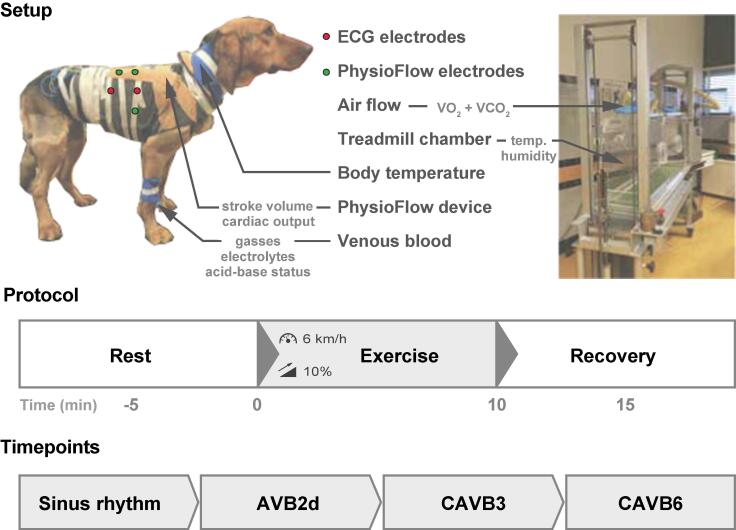
Overview of the experimental design. Setup with overview of techniques and outcome parameters with the dog facing from the right side. Protocol with rest for 5 min, exercise for 10 min at 6 km/h on a 10% inclined treadmill, and recovery for 5 min. Timepoints at which each dog performed the protocol in series: sinus rhythm, and two days (AVB2d), three weeks (CAVB3), and six weeks (CAVB6) after AV block induction.

An overview of the experimental setup to obtain cardiorespiratory parameters is presented in Fig. 1. Four ECG electrodes were placed in a 10–15 cm distance on the back of the dog: two at both sides at the end of the thoracic spine and two on both sides of the start of the lumbar spine. Six lead ECG tracings were recorded with EP tracer (Cardiotek, Maastricht, The Netherlands) at a sampling frequency of 1,000 Hz. For cardiac impedance measurements with the PhysioFlow® (Manatec Biomedical, Petit Ebersviller, Folschviller, France) two electrodes were placed on the left lateral side of the neck, one at the right 3rd intercostal space on the midclavicular line, one at the left 7th intercostal space on the midclavicular line, and two at the back on the level of the xiphoid process of the sternum. The skin at the location of the electrodes was shaved, gel was added between the skin and the electrodes, and vet wrap was used around the electrodes for proper signal detection. Electrodes and cables were connected to the PhysioFlow® device and body weight, length (from the nose to the base of the tail), and the average of three blood pressure measurements from the tail (PetTrust, BioCare, Taoyuan City, Taiwan) were used for automated signal calibration. The detection of each cardiac cycle and the fiducial points of the Z time derivative signal were checked prior to wireless data acquisition using PhysioFlow® software. Venous blood from the cephalic vein in the left paw was collected in a heparin flushed syringe (1 ml) and immediately analyzed (GEM Premier 3000, Werfen, Breda, The Netherlands). pH, pCO_2_ and pO_2_ levels were corrected for body temperature. The treadmill with a custom-build closed chamber of Plexiglass had a continuous flow of 200 l/min and the oxygen uptake (VO_2_) and carbon dioxide exhalation (VCO_2_) within the chamber were measured via a multi-gas analyzer (Servomex Xentra 4100, Almelo, The Netherlands). The temperature and humidity in the chamber were determined with a thermo hygrometer (Testo 635-1, Testo BV, Almere, The Netherlands) and body temperature was obtained by briefly holding the reader to the sensor in the neck of the dog. Cables and the PhysioFlow® device were surrounded by a vest around the thorax and their position was further secured with masking tape. Except for the minimally invasive collection of venous blood, all techniques were non-invasive.

### 2.4 Experimental protocol

Dogs were familiarized with the treadmill, its environment, and the researchers for six weeks before the first experiment was performed to avoid the effect of stress on the outcome parameters. Signs of acquaintance were determined by the researchers based on the known behavior specific to the individual animal and presence of respiratory sinus arrhythmia on the ECG at resting state. The dogs learned to walk on a leash and to run on the treadmill in step with the belt speed by running short periods of exercise (3–5 min, every other day) to avoid intense exercise training. As standard protocol, the dogs were fasted for ± 16 h before the start of each experiment. Surface ECG and PhysioFlow® signals were recorded continuously during the experiment. The temperature, humidity, and VO_2_ and VCO_2_ levels in the empty chamber were documented after which the dog was placed within the treadmill chamber. At rest, the dogs were in a standing position in the treadmill chamber for 5 min, a blood sample and the body temperature were obtained, a VO_2_ and VCO_2_ steady state was reached, and the chamber temperature and humidity were documented. During moderate exercise, the dogs ran for 10 min with a speed of 6 km/h on a 10% inclined treadmill (Fig. 1). They were positively encouraged to walk by cheering and contact between the researchers and the animal was maintained during the experiment. Blood samples, body temperature, VO_2_, VCO_2_, and temperature and humidity in the chamber were obtained during exercise. Next, the treadmill was stopped, and the dogs stayed in the treadmill chamber for 5 min to recover after which a blood sample, body temperature, VO_2_, VCO_2_, chamber temperature and humidity were obtained.

### 2.5 Experimental timepoints

Animals were not randomized in the present study, and operators were not blinded. Every dog performed the experimental protocol in series at four different timepoints serving as their own controls. These include sinus rhythm (SR), 2 days after AV block (AVB2d, 2.2 ± 0.4 days after induction), three weeks after AV block (CAVB3, 3.0 ± 0 weeks after induction) and six weeks after AV block (CAVB6, 6.2 ± 0.4 weeks after induction) (Fig. 1). Remodeling is yet limited at AVB2d, whereas the contractile, electrical, and structural compartments of the AV block-induced cardiac adaptation are completed at CAVB6. In between AVB2d and CAVB3, and CAVB3 and CAVB6, the dogs were brought to the treadmill once a week with a run on the treadmill for 3–5 min to keep acquainted with the experimental environment.

### 2.6 ECG analysis

Electrophysiological parameters were obtained by manually placing time calipers on lead II of the surface ECG using EP tracer software. Intervals of ten beats were averaged and arrhythmic events were excluded. Parameters covered ventricular rate (from RR interval), atrial rate (from PP interval), QT interval, QTc interval (QT interval corrected for heart rate using the Van de Water formula) [19], [20], QRS interval, and JT and JTc interval (QT and QTc corrected for QRS interval, respectively). The occurrence of arrhythmic events in the form of single ectopic beats (sEB) and multiple ectopic beats (mEB), and periods of ventricular tachycardia (VT) were determined during rest (5 min), exercise (10 min) and recovery (5 min).

### 2.7 *PhysioFlow*® *analysis and evaluation*

Stroke volume and cardiac output values were obtained from ten cardiac cycles selected from the exported PhysioFlow® files. The number of cycles depended on the signal quality, QRS morphology and heart rate and therefore 1–9 cycles were available for analysis in a few cases. In addition to the reported evaluation of the PhysioFlow® in conscious beagles [21], the PhysioFlow® was evaluated in mongrel dogs by PV loop recordings, echocardiography, and surface ECG. PV loop tracings were obtained using a 7F pressure catheter in the left ventricle from anesthetized SR dogs laying on the left side (CD Leycom Inc., Hengelo, The Netherlands). Stroke volume was obtained offline from PV loops of 15 selected heart beats via computer software (Conduct NT, CD Leycom). Left ventricular outflow tract (LVOT) and velocity time integral (VTI) from pulsed wave Doppler echocardiograms were used to assess the stroke volume of dogs under anesthesia at SR laying on the left side, and in awake state at SR, AVB2d, and CAVB3 in standing position. Echocardiographic data was analyzed using IntelliSpace Cardiovascular (Philips, Amsterdam, The Netherlands). For data comparison, PhysioFlow® measurements were performed simultaneously with pressure–volume (PV) loop recordings, echocardiography, and surface ECG. Isoprenaline (3 μg/min) was infused at SR and CAVB3 to approach the increase in heart rate and blood pressure during exercise. Under anesthesia, PhysioFlow® electrodes on the back were moved to the xiphoid process of the sternum for proper resting measurements, whereas in awake state they were placed on the back for comparison with measurements during exercise.

### 2.8 Analysis of chamber parameters

VO_2_ and VCO_2_ in ml/min/kg were calculated based on the flow in the chamber (200 l/min), body weight, and the percentage of O_2_ and CO_2_ in the empty chamber and with the dog at steady state (at rest, during exercise, and after recovery). The respiratory exchange ratio (RER) is defined as the ratio of the produced carbon dioxide volume and the consumed oxygen volume during respiration and was calculated by dividing VCO_2_ by VO_2_. The humidity and temperature in the chamber were calculated relative to empty chamber conditions.

### 2.9 Statistical analysis

Data are presented as mean ± standard deviation (SD). Repeated measures two-way analysis of variance (ANOVA) followed by Bonferroni’s or Tukey’s multiple comparisons test were used to analyze group- and timepoint comparisons of serial data. For comparison of two different groups, the unpaired Student’s T test was used. A value of p < 0.05 was considered statistically significant. All statistical analysis were performed using GraphPad Prism (version 9.3.1, GraphPad Software, San Diego, USA).

## 3 Results

### 3.1 AV block dogs with limited remodeling show a lower exercise tolerance

**Fig. 2:**
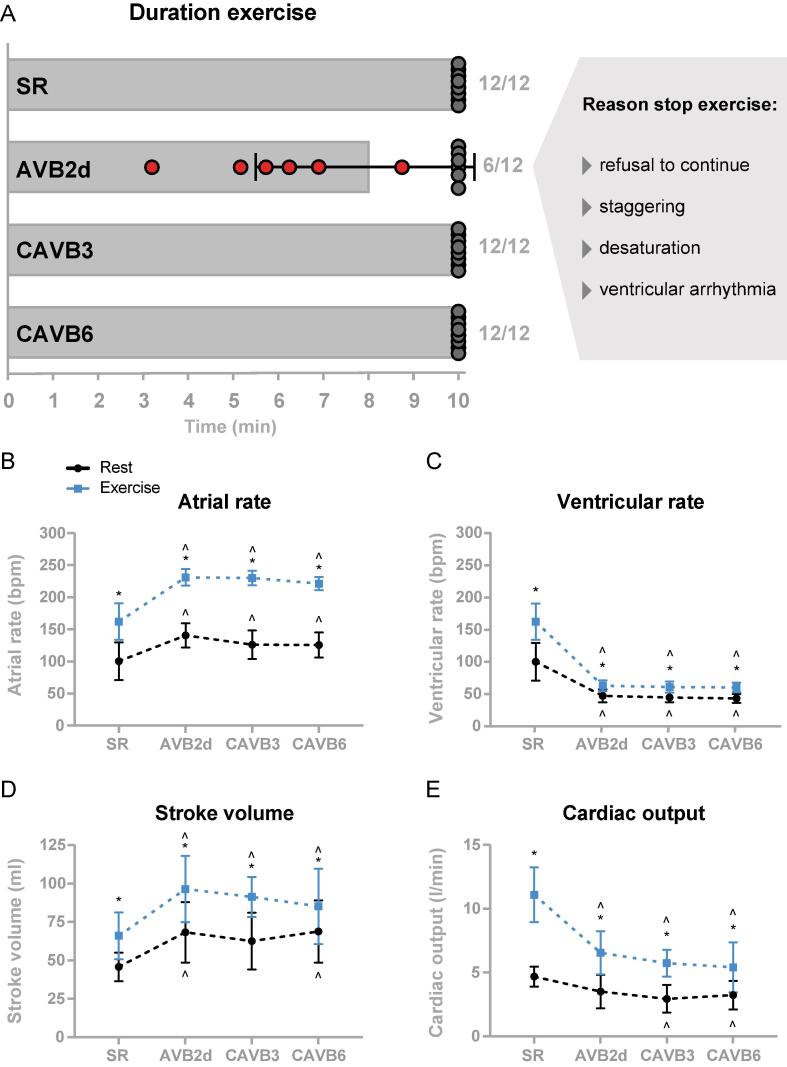
**A)** Exercise duration per experimental timepoint. Six out of twelve dogs at two days after AV block (AVB2d) failed to complete the 10-minute exercise protocol. Those dogs refused to continue the protocol, were staggering on the treadmill, showed signs of hypoxemia, and ventricular arrhythmias (ectopic beats and ventricular tachycardia) occurred. The protocol was completed by all twelve dogs at sinus rhythm (SR), three weeks (CAVB3), and six weeks (CAVB6) after AV block induction. **B)** Atrial rate, **C)** ventricular rate, **D)** stroke volume, and **E)** cardiac output of dogs (n = 12) at rest and during exercise (2 min) at SR, AVB2d, CAVB3, and CAVB6. Data are presented as mean ± SD. For CAVB6 exercise: n = 11, and for stroke volume and cardiac output at AVB2d: n = 10 at rest and n = 9 at exercise. Repeated measures two-way ANOVA with Bonferroni’s multiple comparisons test for comparing rest vs. exercise and Tukey’s multiple comparisons test for comparing SR vs. AV block timepoints. *p < 0.05 compared to rest and ^^^p < 0.05 compared to SR.

The 10-minute moderate exercise protocol was completed by all dogs at SR, CAVB3, and CAVB6. Although the dogs showed signs of fatigue after the exercise, they were visibly able to complete the exercise without refusing to continue while running. However, after repetitively encouragement to continue the exercise protocol, it had to be ended prematurely for 6/12 dogs at AVB2d (Fig. 2**,** red symbols) to avoid a fatal outcome. These animals showed a run duration ranging from 3 min and 12 s to 8 min and 45 s (average run duration of n = 12, mean ± SD: 8 min and 0 s ± 2 min and 24 s). The six dogs that failed the exercise protocol showed more dominant signs of fatigue (panting, head facing forward and stretching the neck for efficient air inhalation, slow response to encouragement, and laying down on the treadmill immediately after stop) compared to SR, CAVB3 and CAVB6. Other reasons of deciding a premature termination of the exercise protocol were a combination of the following: strong refusal to continue (4/6), staggering (5/6), hypoxemia presented by a blue tongue and/or abnormal dark color of the venous blood sample (6/6), vomiting (1/6), and the occurrence of ventricular tachycardia (2/6).

### 3.2 AV block dogs show increased atrial rate after generated idioventricular rhythm

To avoid missing values from dogs unable to complete the exercise protocol, timepoint exercise refers to 2 min of exercise (unless stated otherwise). In SR, exercise increased the atrial and ventricular rate from 100 ± 29 to 162 ± 28 bpm (P < 0.05, Fig. 2**B, C**). Interestingly, the atrial rate was higher at all AV block timepoints compared to SR in rest and it was further increased upon exercise (P < 0.05, Fig. 2**B**). The idioventricular rhythm resulting from AV block induction was significantly lower than the ventricular rate at SR in rest; the rate dropped from 100 ± 29 bpm at SR to 47 ± 10 bpm at AVB2d, 45 ± 8 bpm at CAVB3 and 43 ± 7 bpm at CAVB6 (P < 0.05, Fig. 2**C, Suppl. Table 1**). Exercise increased the ventricular rate at SR with>60 bpm, whereas after AV block this increase was only ± 16 bpm and this was similar at all AV block timepoints. The six dogs that completed the exercise protocol at AVB2d showed a higher ventricular rate upon exercise compared to the six dogs that failed the exercise protocol (P < 0.05, Suppl. Fig. 1**B**). The atrial rate was not different between the completed and failed group. Due to the idioventricular rhythm, the QRS interval was significantly longer at AV block timepoints compared to SR and the interval was not affected by exercise (**Suppl. table 1**). In terms of repolarization, the electrical remodeling induced by AV block is reflected by prolongation of the QT interval corrected for heart rate (QTc) at CAVB3 and CAVB6 (P < 0.05, **Suppl. table 1**). Exercise induced shortening of the QT and JT interval at all four tested timepoints. After AV block, this was predominantly heart rate dependent as the QTc and JTc were unaltered upon exercise.

### 3.3 Exercising dogs are unable to maintain cardiac output after AV block

At rest, stroke volume increased after AV block (P < 0.05, Fig. 2**D, Suppl. table 1**) and exercise increased stroke volume levels at all measured timepoints. Cardiac output was significantly reduced after AV block remodeling at rest at CAVB3 and CAVB6. Exercise highly increased the cardiac output at SR, though less dominant after AV block (P < 0.05, Fig. 2**E, Suppl. table 1**). The six dogs that completed the exercise protocol at AVB2d showed a significant higher stroke volume and a trend towards an increased cardiac output upon exercise compared to the six dogs that failed the exercise protocol (P < 0.05, P = 0.059, Suppl. Fig. 1). A heart weight of 297 ± 33 g and a heart weight/body weight ratio of 11.7 ± 1.0 g/kg were determined after heart isolation at 11 ± 4 weeks of AV block.

The PhysioFlow® technique – used to determine the stroke volume and cardiac output – was evaluated by echocardiography, PV loop measurements and surface ECG, with addition of isoprenaline infusion to simulate a rise in heart rate. This resulted in corresponding heart rate and stroke volume values (Suppl. Fig. 2). The functional adaptation after AV block was confirmed by echocardiography as stroke volume increased after AV block over time (AVB2d and CAVB3).

### 3.4 Proarrhythmia in exercising AV block dogs with limited remodeling

**Fig. 3:**
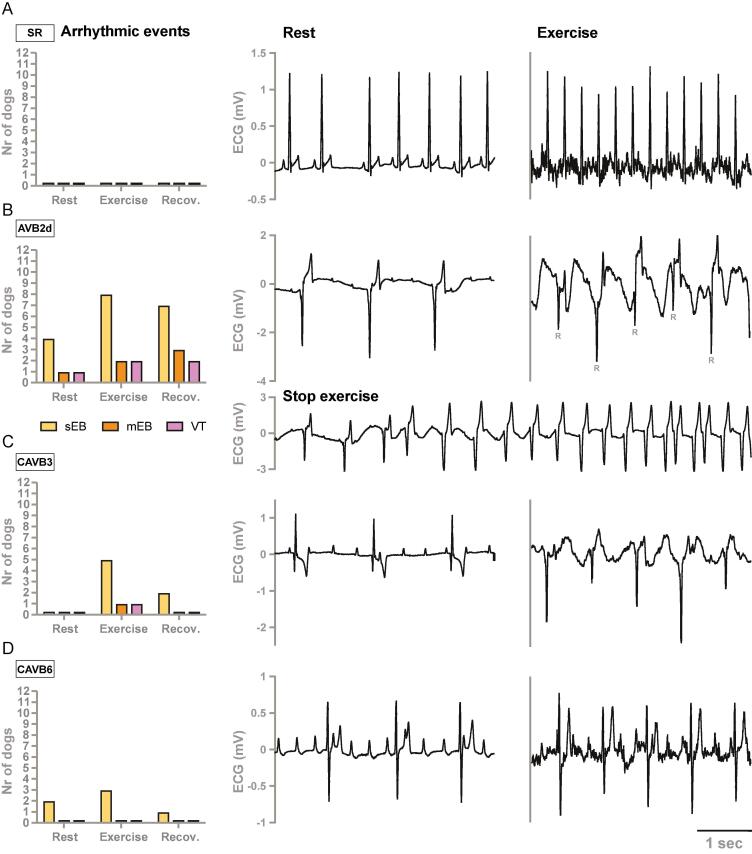
Occurrence of arrhythmic events and representative ECG tracings in dogs (n = 12) at **A)** sinus rhythm (SR), **B)** two days after AV block (AVB2d), **C)** three weeks after AV block (CAVB3) and **D)** six weeks after AV block (CAVB6). Arrhythmic events included single ectopic beats (sEB), multiple ectopic beats (mEB) and ventricular tachycardia (VT) observed at rest (5 min), during exercise (10 min or until premature stop) and recovery period (5 min). Representative ECG tracings of dog #168247 at rest and exercise. This dog stopped the exercise protocol prematurely due to VT, hypoxemia, and staggering.

Dogs at SR showed no arrhythmic events during the experimental protocol (Fig. 3**A**). Arrhythmic events were mostly present in dogs at AVB2d (Fig. 3**B**). Already at rest, four dogs showed EBs and exercise further induced the occurrence of EBs, mEBs and VT. Of the six dogs that failed the exercise protocol at AVB2d, five dogs showed arrhythmic events during exercise. Arrhythmic events were also present at CAVB3 and CAVB6, yet less dominant (Fig. 3**C, D**). Moreover, QRS morphology changes - reflecting origin variations of the ventricular pacemaker focus - were predominantly present at AVB2d and the origin was stabilized at CAVB3 and CAVB6 over time. Representative ECG tracings obtained during the experimental protocol illustrate the presence of respiratory sinus arrhythmia in resting dogs at SR, the increase in ventricular rate during exercise, and the specific characteristic of AV block by the absence of a relation between the P waves and the QRS complexes (Fig. 3). Furthermore, the occurrence of a VT is presented in a dog at AVB2d that stopped the exercise protocol prematurely.

### 3.5 Exercising dogs with limited remodeling have a lower oxygen uptake

**Fig. 4:**
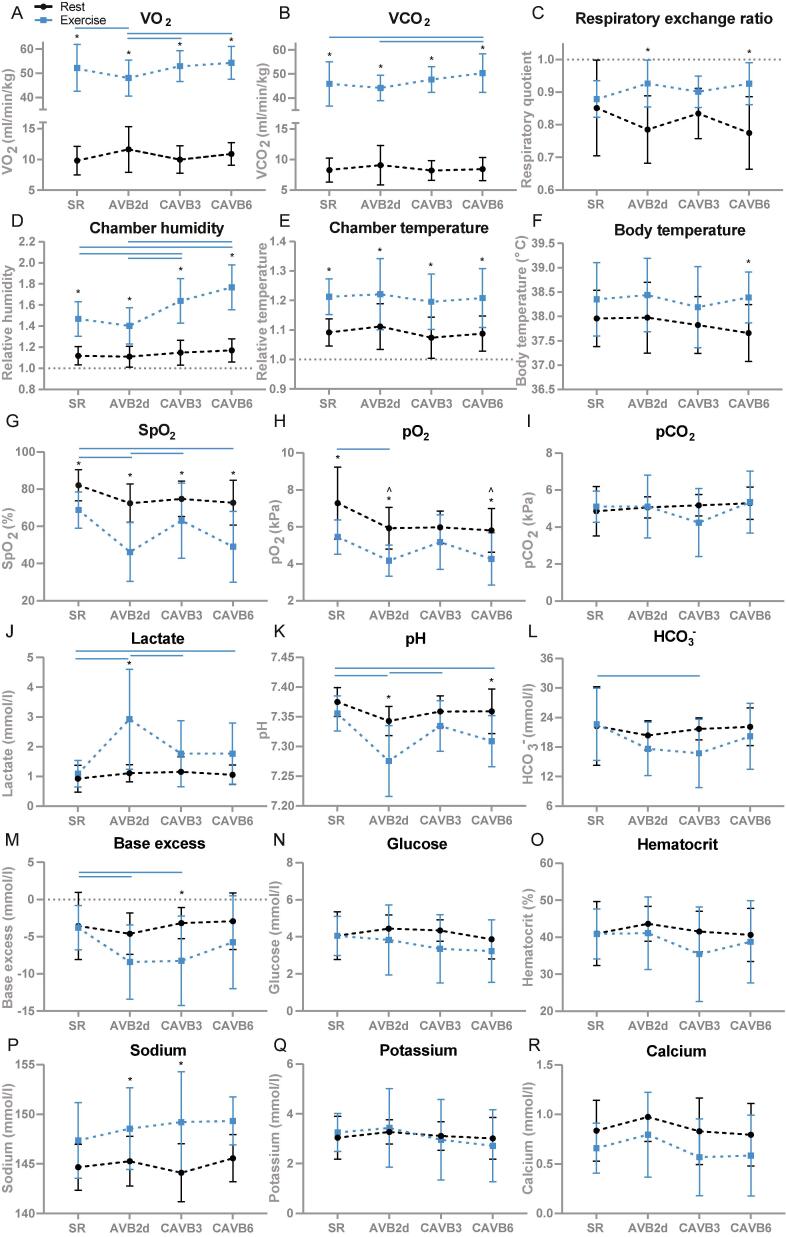
**A)** Oxygen uptake (VO_2_), **B)** carbon dioxide exhalation (VCO_2_), **C)** respiratory exchange ratio, **D)** chamber humidity, **E)** chamber temperature and **F)** body temperature of dogs (n = 12) at rest and during exercise (10 min or steady state when the exercise protocol was stopped prematurely) at sinus rhythm (SR), and two days (AVB2d), three weeks (CAVB3) and six weeks (CAVB6) after AV block induction. Chamber humidity and temperature are relative to empty chamber conditions. Data are presented as mean ± SD, with n = 10–12. Repeated measures two-way ANOVA with Bonferroni’s multiple comparisons test for comparing rest vs exercise and Tukey’s multiple comparisons test for comparing SR vs. AV block timepoints. *p < 0.05 compared to rest and blue bars refer to p < 0.05 in exercise group. **G-R)** Venous blood parameters of dogs at rest and exercise (2 min) at SR, AVB2d, CAVB3, and CAVB6 after AV block induction. Data are presented as mean ± SD. For rest: SR n = 12, AVB2d n = 11, CAVB3 n = 9 and CAVB6 n = 7–11. For exercise: SR n = 11, AVB2d n = 9, CAVB3 n = 9 and CAVB6 n = 6–10. Repeated measures two-way ANOVA with Bonferroni’s multiple comparisons test for comparing rest vs. exercise and Tukey’s multiple comparisons test for comparing SR vs. AV block timepoints. *p < 0.05 compared to rest/exercise, ^^^p < 0.05 compared to SR and blue bars refer to p < 0.05 in exercise group. (For interpretation of the references to color in this figure legend, the reader is referred to the web version of this article.)

At rest, VO_2_ and VCO_2_ were not different after AV block (Fig. 4**A, B**). Exercise increased the VO_2_ and VCO_2_ significantly in dogs at SR and all AV block timepoints. The VO_2_ in dogs at AVB2d was significantly lower upon exercise compared to SR, CAVB3 and CAVB6 (P < 0.05, Fig. 4**A**). For VCO_2_, values were lower at AVB2d compared to CAVB6 and higher at CAVB6 compared to SR (P < 0.05, Fig. 4**B**). The RER, a parameter reflecting the fuel selection during exercise based on VCO_2_ and VO_2_, was not altered after AV block at rest and was increased during exercise at AVB2d and CAVB6 compared to rest (Fig. 4**C**). The humidity in the treadmill chamber relative to empty conditions was elevated due to exercise (**Suppl. table 1**), which was more pronounced at CAVB3 and CAVB6 (Fig. 4**D**). Exercise increased the temperature in the treadmill chamber at all measured timepoints (P < 0.05, Fig. 4**E, Suppl. table 1**), and apart from CAVB6 the body temperature was unaffected at rest and exercise (Fig. 4**F**).

### 3.6 Hypoxemia and acidification in exercising AV block dogs with limited remodeling

Venous blood was analyzed to determine if the exercise-induced cardiorespiratory changes were reflected in the venous blood parameters. Upon exercise, dogs showed reduced SpO_2_ levels at all timepoints and diminished pO_2_ levels at SR, AVB2d and CAVB6 (P < 0.05, Fig. 4**G, H**). Oxygen desaturation was mostly present in exercising dogs at AVB2d, as SpO_2_ levels were lowest at that timepoint and significantly different from SR and CAVB3. Moreover, pO_2_ levels were significantly lower compared to SR in resting and exercising dogs at AVB2d.

Venous blood from exercising dogs at AVB2d was more acidic as demonstrated by an increase in lactate, and a lower pH and base access (Fig. 4**J, K, M**). The latter parameter and HCO_3_^−^ were reduced in exercising dogs at CAVB3 compared to SR (P < 0.05, Fig. 4**M, L**). Dogs at CAVB6 showed the following alterations in venous blood parameters: exercise reduced SpO_2_ and pH, and increased lactate levels compared to SR. However, these values were not different from other AV block timepoints. In terms of electrolytes, sodium levels were significantly higher upon exercise in dogs at AVB2d and CAVB3 (P < 0.05, Fig. 4**P**). Exercise and AV block did not significantly affect pCO_2_, glucose, hematocrit, potassium, and calcium levels (Fig. 4**I, N, O, Q, R**).

## 4 Discussion

### 4.1 The exercising AV block dog model

The most frequently used exercise protocol in the clinic is the Bruce treadmill test which includes gradual changes in speed and a slope increase of 22% to assess aerobic exercise capacity [22]. For diseased children, these maximal treadmill protocols at a high incline can be too demanding and therefore individualized protocols were examined [23]. To avoid early loss of our included animals due to a too demanding exercise protocol after the atrioventricular intervention, a constant workload treadmill protocol for 10 min at a 10% incline was used in the current study. The protocol was considered moderate as the heart rate of dogs at SR increased to ± 55% of the reported maximal heart rate upon exercise [24], [25].

Chronic complete heart block in dogs by a direct incision of the His bundle was first developed as a model of experimental heart failure confirmed by e.g. low exercise tolerance on a treadmill [26]. Later, treadmill experiments in dogs with AV block focused on improvement of cardiac output by pacing [27] and hormone responses to local wall stress [28]. The time course after AV block varied from one week to several months without detailed evaluation of AV block-induced cardiac adaptations, which would highly affect outcome. In the current study, the evaluated timepoints after AV block at AVB2d, CAVB3 and CAVB6 reflect the different remodeling stages that evolve after AV block induction. These include limited remodeling at the early stage, stable contractile and electrical remodeling after two weeks, and stable structural remodeling after four to six weeks [11]. With minimal to non-invasive techniques, we determined cardiorespiratory changes upon exercise at the different remodeling stages. Exercise induced significant increases in atrial and ventricular rate, stroke volume, cardiac output, VO_2_, VCO_2_, chamber humidity and temperature, and significant decreases in venous SpO_2_ and pO_2_ levels. Evaluation of cardiac output includes predominantly invasive methods, though the non-invasive PhysioFlow® system based on cardiac impedance was previously evaluated in conscious beagle dogs [21]. Here, evaluation of the system in anesthetized and awake mongrel dogs, including simulation of increases in heart rate by infusion of isoprenaline, resulted in reliable measures of heart rate and stroke volume when evaluated by ECG, echocardiography, and PV loop analyses. The Physioflow® is considered as a reliable and non-invasive technique to measure cardiac output in awake mongrel dogs.

### 4.2 AV block-induced remodeling and exercise tolerance

Shortening of the PP interval directly after AV block was already reported before [29] and is explained by the enhanced adrenergic activity in order to compensate for the bradycardia-induced drop in cardiac output [13]. This response was further augmented upon exercise (atrial rate > 230 bpm) while the ventricular rate only increased to 66 bpm. Unpaced children with AV block are able to increase their ventricular rate upon maximal exercise to 117 ± 48 bpm [30]. The difference in exercise protocol – being moderate in our study – likely explains the lower increase in ventricular rate in our study. The increase in cardiac output during exercise is mainly dependent on the increase in heart rate and partly on the increase in stroke volume [31]. Indeed, the dogs that completed the exercise protocol at AVB2d had a higher ventricular rate. Moreover, their stroke volume was higher than those that failed the exercise protocol, all resulting in a higher cardiac output upon exercise. These results were also found in children with AV block, as the unpaced children showed similar VO_2peak_ levels as paced children [30]. It was suggested that only the ‘best’ patients stay unpaced. Furthermore, it was implied that children with AV block generate more energy from anaerobic sources during exercise as compensatory mechanism since the peak workload was similar to healthy peers [30]. For the dogs at AVB2d, this can be confirmed by the venous blood parameters showing signs of acidification by a buildup of lactate and the corresponding reduction in venous pH, and hypoxemia by the reduced venous SpO_2_ and pO_2_. Certainly, these conditions have resulted in premature termination of the exercise protocol as was decided when the dogs at AVB2d were staggering, showed a desaturated tongue and convincingly refused to continue the exercise. Another reason of premature termination was the occurrence of ventricular tachycardia. Indeed, in addition to the known changes in origin of ventricular pacemaker focus, the dogs at AVB2d showed more arrhythmic events upon exercise. The hypoxic conditions at this timepoint could have contributed to myocardial ischemia resulting in abnormal automaticity and the thereby induced arrhythmic events [32].

Despite the high dependence of cardiac output on heart rate, stroke volume must increase to a certain level to compensate for the severe bradycardia after AV block in order to maintain cardiac output levels similar to SR. Previous studies showed that AV block dogs with chronic remodeling under anesthetized conditions increased their cardiac output almost to SR conditions due to enhanced inotropic parameters [15], [33]. In the current study, cardiac hypertrophy induced by AV block was confirmed as the heart weight and heart weight/body weight values coincide with previously reported results [13]. We also found that the cardiac output was lowest at CAVB3 and CAVB6 at rest in awake conditions, and during exercise it was lower than SR at all AV block timepoints, while all dogs at CAVB3 and CAVB6 completed the exercise protocol. An explanation could be that, in contrast to AVB2d, dogs at CAVB3 and CAVB6 showed a similar steady state VO_2_ as at SR. Besides the cardiac remodeling and increased neurohormonal parameters after AV block, a role for remodeling of other organs/tissues important for exercise is implied here. In the AV block dog, chronic remodeling may also include optimization of respiratory ventilation, blood flow regulation, and O_2_ extraction in the active muscle which all affect the VO_2_ [34].

### 4.3 The exercising AV block dog model: A translational perspective

Software with self-adapting computational neurons based on feedback of various physiological parameters, such as blood pressure, SpO_2_, and respiration can be the basis of new cardiac rhythm management [7]. The use of a biofeedback device in cardiac pacing on the respiratory cycle resulted in an improved cardiac output in sheep with heart failure [35]. We suggest that the induction of cardiorespiratory changes upon exercise in the preclinical exercising AV block dog model can be used to test and optimize new device therapies. Improvement of cardiac function and thereby exercise tolerance due to new cardiac device properties can be determined at AVB2d, when the animal’s exercise tolerance is most vulnerable.

### 4.4 Study limitations

The lower detection limit of heart rate by the PhysioFlow® was 40 bpm for calibration at resting state resulting in the exclusion of two measurements. The best parameter to describe exercise capacity is VO_2peak_, though, this study does not include invasive measures of arterial blood and therefore the arteriovenous oxygen difference as part of the VO_2peak_ (according to the Fick equation) could not be determined. The individual remodeling stages over time were not confirmed in the animals included in this study. Moreover, the heterogeneity in activity behavior between animals can represent the variation of human activity in daily life, however, the exact effect of the daily activity in the stables on the experimental outcome cannot be excluded.

### 4.5 Conclusion

In the present study, it was observed that dogs with limited remodeling after AV block at AVB2d have a lower exercise tolerance than dogs with remodeling at three and six weeks after AV block induction. Moreover, the bradycardia-induced adaptations at all AV block timepoints are an increased atrial rate and stroke volume, though these electrical and functional adjustments fail to completely maintain the cardiac output levels as seen at regular sinus rate. The results further demonstrate that exercising dogs at AVB2d show more arrhythmic events, a lower VO_2_, and signs of hypoxemia and acidification.

## CRediT authorship contribution statement

**Joanne J.A. van Bavel:** Conceptualization, Formal analysis, Investigation, Methodology, Validation, Visualization, Writing – original draft, Writing – review & editing. **Henriëtte D.M. Beekman:** Investigation. **Arend Schot:** Investigation. **Philippe C. Wouters:** Investigation. **Maarten G. van Emst:** Conceptualization. **Tim Takken:** Conceptualization, Resources. **Marcel A.G. van der Heyden:** Supervision, Writing – review & editing. **Marc A. Vos:** Conceptualization, Supervision.

## Declaration of Competing Interest

The authors declare that they have no known competing financial interests or personal relationships that could have appeared to influence the work reported in this paper.
