# Supplementary material for: Remodeling in the AV block dog is essential for tolerating moderate treadmill activity
Source: Int J Cardiol Heart Vasc. 2023 Jan 3;44:101169. doi: 10.1016/j.ijcha.2022.101169 (PMC9826816; doi:10.1016/j.ijcha.2022.101169)
Supplement: Supplementary data 1 [file mmc1.docx]

**Remodeling in the AV block dog is essential for tolerating moderate treadmill activity**

Joanne J.A. van Bavel^1^, Henriëtte D.M. Beekman^1^, Arend Schot^2^, Philippe C. Wouters^3^, Maarten G. van Emst^2^, Tim Takken^4^, Marcel A.G. van der Heyden^1,^*, Marc A. Vos^1^

^1^ Department of Medical Physiology, ^3^ Department of Cardiology, Division of Heart & Lungs, University Medical Center Utrecht, Utrecht, The Netherlands

^2^ Department of Clinical Sciences, Division of Anatomy and Physiology, Faculty of Veterinary Medicine, Utrecht University, The Netherlands

^4^ Child Development and Exercise Center, Wilhelmina Children’s Hospital, University Medical Center Utrecht, Utrecht, The Netherlands

**Supplemental data**

**Suppl. table 1.** Electrophysiological parameters, stroke volume, cardiac output, chamber humidity and temperature from dogs (n=12) at sinus rhythm (SR) and two days (AVB2d), three weeks (CAVB3) and six weeks (CAVB6) after AV block induction at rest, during exercise (2, 5, and 10 minutes) and 5 minutes of recovery (recov.).

|  |  | **Rest** | **2 min** | **5 min** | **10 min** | **Recov.** |
| --- | --- | --- | --- | --- | --- | --- |
| Atrial rate | SR | 100 ± 29 | 162 ± 28* | 167 ± 18* | 176 ± 16* | 114 ± 23 |
|  | AVB2d | 141 ± 19^ | 231 ± 13*^ | 220 ± 27*^ | 223 ± 17*^ | 166 ± 12*^ |
|  | CAVB3 | 126 ± 22^ | 230 ± 11*^ | 233 ± 11*^ | 239 ± 12*^ | 148 ± 24*^ |
|  | CAVB6 | 126 ± 20^ | 221 ± 10*^^#^ | 224 ± 12*^^#^ | 229 ± 13*^^#^ | 137 ± 20^^&^ |
| Ventricular rate | SR | 100 ± 29 | 162 ± 28* | 167 ± 18* | 176 ± 16* | 114 ± 23 |
|  | AVB2d | 47 ± 10^ | 63 ± 9*^ | 66 ± 15*^ | 66 ± 10*^ | 45 ± 11^ |
|  | CAVB3 | 45 ± 8^ | 61 ± 9*^ | 63 ± 8*^ | 64 ± 7*^ | 43 ± 7^ |
|  | CAVB6 | 43 ± 7^ | 60 ± 8*^ | 62 ± 7*^ | 63 ± 9*^ | 40 ± 6^ |
| QRS | SR | 73 ± 6 | 67 ± 5 | 67 ± 6 | 67 ± 6 | 72 ± 8 |
|  | AVB2d | 96 ± 9^ | 87 ± 6^ | 87 ± 5^ | 87 ± 8^ | 94 ± 9^ |
|  | CAVB3 | 103 ± 13^ | 93 ± 13^ | 96 ± 12^ | 95 ± 11^ | 100 ± 12^ |
|  | CAVB6 | 98 ± 11^ | 99 ± 12^ | 101 ± 12^^&^ | 104 ± 12^^&^ | 101 ± 12^ |
| QT | SR | 234 ± 24 | 196 ± 21* | 192 ± 11* | 189 ± 11* | 222 ± 16* |
|  | AVB2d | 296 ± 24^ | 261 ± 20*^ | 256 ± 16*^ | 253 ± 25*^ | 288 ± 28^ |
|  | CAVB3 | 338 ± 35^^&^ | 285 ± 20*^^&^ | 281 ± 26*^^&^ | 281 ± 22*^^&^ | 325 ± 35^^&^ |
|  | CAVB6 | 332 ± 30^^&^ | 300 ± 25*^^&^ | 298 ± 29*^^&^ | 294 ± 28*^^&^ | 332 ± 25^^&^ |
| QTc | SR | 265 ± 15 | 250 ± 15* | 247 ± 9* | 246 ± 9* | 261 ± 10 |
|  | AVB2d | 269 ± 19 | 264 ± 19^ | 261 ± 18 | 259 ± 24 | 254 ± 21 |
|  | CAVB3 | 306 ± 28^^&^ | 285 ± 15^^&^ | 284 ± 18^^&^ | 286 ± 18*^ | 287 ± 25*^^&^ |
|  | CAVB6 | 296 ± 24^ | 300 ± 17^^&^ | 299 ± 25^^&^ | 297 ± 21^^&^ | 286 ± 18^^&^ |
| JT | SR | 161 ± 19 | 128 ± 18* | 125 ± 12* | 122 ± 11* | 150 ± 13 |
|  | AVB2d | 201 ± 23^ | 173 ± 19*^ | 169 ± 16*^ | 166 ± 27*^ | 193 ± 24^ |
|  | CAVB3 | 235 ± 25^^&^ | 192 ± 17*^^&^ | 185 ± 19*^ | 186 ± 15*^ | 225 ± 25^^&^ |
|  | CAVB6 | 234 ± 24^^&^ | 201 ± 17*^^&^ | 196 ± 22*^^&^ | 190 ± 20*^ | 230 ± 19^^&^ |
| JTc | SR | 193 ± 12 | 182 ± 13 | 181 ± 11* | 179 ± 9* | 189 ± 9 |
|  | AVB2d | 173 ± 17^ | 176 ± 16 | 173 ± 14 | 172 ± 23 | 160 ± 20^ |
|  | CAVB3 | 203 ± 19^&^ | 192 ± 11^&^ | 188 ± 12^&^ | 190 ± 10 | 187 ± 19^&^ |
|  | CAVB6 | 198 ± 18 | 201 ± 12^^&^ | 198 ± 18^^&^ | 192 ± 14^ | 184 ± 15^&^ |
| Stroke volume | SR | 46 ± 9 | 66 ± 15* | 65 ± 16* | 66 ± 21* | 49 ± 10 |
|  | AVB2d | 68 ± 20^ | 96 ± 21*^ | 95 ± 21*^ | 94 ± 12 | 70 ± 25 |
|  | CAVB3 | 63 ± 18^ | 91 ± 13*^ | 89 ± 16*^ | 89 ± 21*^ | 56 ± 20 |
|  | CAVB6 | 69 ± 24^ | 85 ± 24 | 88 ± 22*^ | 86 ± 21* | 69 ± 23^ |
| Cardiac output | SR | 4.7 ± 0.8 | 11.1 ± 2.2* | 11.2 ± 3.2* | 11.6 ± 3.8* | 5.5 ± 1.1 |
|  | AVB2d | 3.5 ± 1.3 | 6.5 ± 1.7*^ | 6.4 ± 1.6*^ | 6.9 ± 1.0 | 3.7 ± 1.6 |
|  | CAVB3 | 2.9 ± 1.1^ | 5.7 ± 1.1*^ | 5.7 ± 1.4*^ | 5.8 ± 1.7*^ | 2.6 ± 1.2^ |
|  | CAVB6 | 3.2 ± 1.1^ | 5.4 ± 1.9*^ | 5.7 ± 1.7*^ | 5.7 ± 1.8*^ | 3.1 ± 1.1^ |
| Chamber humidity | SR | 44.5 ± 3.9 | 52.0 ± 7.1* | 55.7 ± 10.3* | 58.6 ± 9.7* | 52.1 ± 8.2* |
|  | AVB2d | 42.6 ± 4.3 | 50.4 ± 5.9* | 54.1 ± 7.4* | 54.7 ± 7.4* | 47.4 ± 6.2 |
|  | CAVB3 | 43.7 ± 4.4 | 51.7 ± 5.1* | 57.1 ± 7.6* | 61.3 ± 7.7* | 50.0 ± 6.6 |
|  | CAVB6 | 41.1 ± 4.5 | 52.5 ± 7.2* | 57.2 ± 9.5* | 63.0 ± 6.8* | 47.6 ± 7.2 |
| Chamber temperature | SR | 19.5 ± 2.2 | 20.1 ± 2.2* | 20.7 ± 2.0* | 21.6 ± 1.9* | 21.3 ± 1.9* |
|  | AVB2d | 19.9 ± 2.8 | 20.4 ± 2.8* | 20.7 ± 2.5* | 21.6 ± 2.4* | 21.3 ± 2.4* |
|  | CAVB3 | 19.8 ± 1.1 | 20.2 ± 1.0* | 20.9 ± 1.1* | 21.8 ± 0.9* | 21.5 ± 1.0* |
|  | CAVB6 | 19.9 ± 1.8 | 20.6 ± 1.9* | 21.4 ± 1.9* | 22.2 ± 1.6* | 21.9 ± 1.7* |

QTc and JTc were obtained by QT and JT correcting for heart rate using the Van de Water formula. Ventricular and atrial rate in beats per minute, ECG intervals in milliseconds, stroke volume in ml, cardiac output in l/min, chamber humidity in %, and chamber temperature in °C. Data are presented as mean ± SD. For AVB2d exercise: n=8-12, for CAVB6 exercise: n=11, and for stroke volume and cardiac output at AVB2d: n=10 at rest and n=4-9 at exercise, and at CAVB6: n=11. Repeated measures two-way ANOVA with Tukey’s multiple comparisons test. *p<0.05 compared to rest, ^p<0.05 compared to SR, ^&^p<0.05 compared to AVB2d, and ^#^p<0.05 compared to CAVB3.

**Suppl. figure 1.** **A)** Atrial rate, **B)** ventricular rate, **C)** stroke volume, and **D)** cardiac output of dogs at timepoints rest (left panel) and exercise (right panel) separated in the failed (red, n=6) and completed (black, n=6) group determined at AVB2d. Data are presented as mean ± SD. For the completed group at AVB2d, parameters stroke volume and cardiac output at exercise: n=3. Unpaired t-test for comparison of failed vs completed group, *p<0.05.

**Suppl. figure 2.** PhysioFlow evaluation in dogs by **A)** heart rate and **B)** stroke volume at sinus rhythm (SR) under anesthesia (anesth.), and in awake state at SR, two days (AVB2d) and three weeks (CAVB3) after AV block induction in baseline or after isoprenaline (iso). PhysioFlow data (in blue) was compared to echocardiography (echo, in black), pressure-volume loop measurements (PV-loop, in red), and electrocardiogram (ECG, in yellow). Data (numbers below data symbols refer to group size) are presented as mean ± SD.
